# Case Report: Superficial spreading squamous cell carcinoma of the cervix: a retrospective single-center case series and review of the literature

**DOI:** 10.3389/fonc.2026.1829547

**Published:** 2026-10-21

**Authors:** Hui-qiong Huang, Feng-ming Gong, Yu Xuan, Xiao-xia Wei, Xuan Zuo

**Affiliations:** 1 Department of Oncology Radiotherapy and Chemotherapy, West China Second University Hospital, Chengdu, China; 2 Key Laboratory of Birth Defects and Related Diseases of Women and Children, Ministry of Education, Sichuan University, Chengdu, China; 3 Department of Pathology, West China Second University Hospital, Chengdu, China

**Keywords:** cervical cancer, diagnostic criteria, literature review, retrospective case series, superficial spreading squamous cell carcinoma

## Abstract

**Background:**

Superficially spreading squamous cell carcinoma of the cervix (ss-SCC) is a rare growth pattern characterized by predominantly mucosal extension of a cervical squamous lesion along the genital tract. Its diagnostic criteria, prognostic significance, and implications for staging and management remain uncertain. This study aimed to establish reproducible pathological criteria, characterize clinicopathological features and outcomes, and summarize the available literature.

**Methods:**

We retrospectively reviewed cases diagnosed at our institution between January 2010 and December 2025. Inclusion required a cervical-centered squamous lesion with histologically demonstrable superficial continuity with at least one extra-cervical genital-tract site, predominantly mucosal growth, morphological concordance between involved sites, and exclusion of metastatic neoplasm involving the cervix. All cases were reassessed according to the 2020 WHO classification and staged using the 2018 FIGO system. Clinical, pathological, treatment, recurrence, and follow-up data were summarized descriptively. PubMed was searched through December 31, 2025 using predefined eligibility criteria.

**Results:**

Seven patients (mean age, 60 years; range, 50–77 years) met the diagnostic criteria. All tumors showed continuous superficial epithelial lesions extention from the cervix to the endometrium, with additional vaginal or fallopian-tube involvement in selected cases. All cervical lesions contained an invasive SCC component, with a maximum measured stromal invasion depth no greater than 5 mm; background HSIL was documented in four cases. All seven cases showed block-type p16 expression in the cervical and extra-cervical components. During a median follow-up of 30 months (range, 11–54 months), two patients developed locoregional recurrence (2/7, 28.6%). Neither had received initial adjuvant therapy, and both underwent salvage chemoradiotherapy and were alive without evidence of disease at the last follow-up. No disease-related deaths occurred. The literature review identified 25 publications comprising 44 patients, but heterogeneous treatment and incomplete follow-up precluded pooled outcome analysis.

**Conclusion:**

ss-SCC represents a distinctive histological growth pattern within the HPV-associated cervical SCC spectrum rather than a separately staged entity. Diagnosis requires confirmation of cervical origin, demonstration of continuous superficial mucosal extension, and distinction from alternative primary tumors or conventional invasive/metastatic disease. Pathology reports should document the extent and distribution of mucosal involvement, invasion depth at each involved site, LVSI, and margin status. Multifocal anatomical involvement, including superficial mucosal extension, was not consistently associated with adverse outcomes in the reported cases. However, this conclusion requires further validation in larger case series with longer-term follow-up.

## 1 Introduction

The 2020 WHO Classification of Female Genital Tumors adopts a two-tiered system for HPV-associated squamous intraepithelial lesions, classifying them as low-grade squamous intraepithelial lesion (LSIL) and high-grade squamous intraepithelial lesion (HSIL). The historical terminology of cervical intraepithelial neoplasia (CIN 1–3) and carcinoma *in situ* (CIS) is retained only when referring to previously published studies or harmonizing historical pathological diagnoses (1). Invasive cervical squamous cell carcinoma (SCC) is classified according to HPV association and staged anatomically using the 2018 FIGO staging system and the AJCC TNM classification. For FIGO stage IA disease, stromal invasion must be microscopically measured: IA1 is defined as invasion ≤3 mm in depth, whereas IA2 is defined as invasion >3 mm but ≤5 mm. Lymphovascular space invasion (LVSI) should be documented because of its prognostic and therapeutic implications, although its presence alone does not modify FIGO stage (1–4). The conventional progression model of cervical SCC involves sequential transition from intraepithelial neoplasia to destructive stromal invasion, followed by potential direct extension and/or lymphatic or hematogenous dissemination (5). In contrast, superficially spreading squamous cell carcinoma of the cervix (ss-SCC) represents an uncommon growth pattern characterized by a cervical-centered squamous lesion with predominant superficial mucosal extension along the contiguous genital tract, while stromal invasion within the cervix is absent, minimal, or otherwise disproportionate to the extent of mucosal spread. Historically, this pattern has been described using heterogeneous terminology, including superficial extension of CIN/CIS and superficially spreading cervical SCC, reflecting the lack of standardized diagnostic criteria.

Importantly, ss-SCC is not currently recognized as a distinct category in the 2020 WHO classification and is not assigned a dedicated staging framework in either the FIGO or AJCC systems (1, 2, 4). Therefore, the biological significance of superficial extra-cervical mucosal involvement, its distinction from conventional invasive spread, and its implications for clinical management remain incompletely defined. In this study, we reviewed seven institutionally diagnosed cases and analyzed the published literature to establish reproducible diagnostic criteria, characterize clinicopathological features and outcomes, and propose practical considerations for pathological reporting and multidisciplinary management.

## 2 Materials and methods

### 2.1 Case retrieval and data collection

The pathology database of West China Second University Hospital, Sichuan University, was retrospectively searched for cases diagnosed between January 2010 and December 31, 2025. Case identification was performed using historical and current diagnostic terms, including cervical carcinoma *in situ* (CIS), microinvasive squamous cell carcinoma (SCC), high-grade squamous intraepithelial lesion (HSIL), and invasive SCC, with additional screening for cases showing squamous lesions involving contiguous extra-cervical genital tract sites, including the endometrium, fallopian tubes, ovaries, or vagina. Historical terminology was used solely for archival case retrieval, whereas final diagnoses and pathological assessments were based on the current WHO classification.

For potentially eligible cases, available hematoxylin and eosin (H&E)-stained slides, pathology reports, clinical records, imaging findings, treatment information, recurrence status, and follow-up data were systematically reviewed. After histopathological reassessment according to the predefined operational criteria, seven cases were confirmed to meet the diagnostic requirements for ss-SCC.

### 2.2 Pathological diagnostic criteria and review of H&E sections

All available histological slides from cases diagnosed between 2010 and 2025 were re-evaluated by a gynecological pathologist and classified according to the fifth edition (2020) WHO Classification of Female Genital Tumors. Tumor staging was assigned according to the 2018 FIGO staging system (1, 2). Current WHO terminology was applied to all cases in the institutional cohort. Historical diagnostic terms were retained only when necessary to describe terminology used in previously published literature.

The histopathological criteria applied for case reassessment were as follows:

• Cervical HSIL: A high-grade squamous intraepithelial lesion of the cervix showing HPV-associated atypical squamous proliferation involving the surface epithelium and/or endocervical glandular epithelium, without definite stromal invasion beyond the epithelial basement membrane. Historical diagnoses of CIN 2, CIN 3, or carcinoma *in situ* were reassigned as HSIL according to the current WHO classification.
• Conventional invasive cervical SCC: A malignant squamous neoplasm characterized by destructive stromal infiltration of tumor cells beyond the epithelial basement membrane, typically with an infiltrative growth pattern and an associated stromal desmoplastic or inflammatory response. According to the 2020 WHO Classification of Female Genital Tumors, unequivocal stromal invasion defines the transition from squamous intraepithelial lesion to invasive carcinoma. The depth of stromal invasion is measured microscopically in millimeters and used for FIGO staging, with stage IA1 defined as invasion ≤3 mm and stage IA2 as invasion >3 mm but ≤5 mm according to the 2018 FIGO staging system. Lymphovascular space invasion (LVSI), margin status, tumor size, and other relevant pathological parameters should be documented separately because of their prognostic and clinical management implications (1, 2).

Operational definition of ss-SCC. For inclusion in this series, all of the following criteria were required:

• Cervical origin: A morphologically identifiable lesion centered in the cervix.
• Superficial anatomical continuity: Continuous or directly traceable replacement of the mucosal or glandular epithelium from the cervix to at least one extra-cervical genital-tract site across the submitted histological sections.
• Predominantly mucosal growth: The extra-cervical component was predominantly intraepithelial or confined to superficial mucosa; limited stromal invasion at the distal site was acceptable when interpreted as a continuation of the same tumor process.
• Exclusion of an alternative primary: The entire cervix should be submitted for histopathological examination. No independent squamous carcinoma was identified elsewhere in the genital tract, particularly in the vagina. Spatially separate squamous lesions lacking demonstrable anatomical continuity were excluded.
• Morphological concordance: The cervical and extra-cervical components demonstrated concordant squamous morphology. Immunophenotypic concordance, including block-type p16 expression when available, was considered supportive evidence.

### 2.3 Immunohistochemistry (IHC)

ER and PR immunohistochemistry was performed as an exploratory analysis to investigate the potential association between postmenopausal status and the ss-SCC growth pattern. Neither marker was used for the diagnosis of cervical squamous neoplasia or for determining the primary site of origin. The comparison group consisted of five patients with cervix-confined HSIL from reproductive-aged individuals. Representative cases of cervix-confined invasive SCC and non-neoplastic cervical squamous epithelium were included only as illustrative tissue controls and were not considered formal comparator cohorts.

P16 immunohistochemistry was evaluated in both cervical and extra-cervical components of all seven ss-SCC cases as a surrogate marker of an HPV-associated squamous phenotype; however, p16 expression alone was not interpreted as evidence of clonality or proof of common tumor origin. CK5/6 immunostaining was performed in Case 7 to support squamous differentiation of the adnexal lesion and was interpreted as an ancillary finding.

**Table:** Antibody sources and application details.

Antibody	Clone number	Type	Running platform
ER	SP1	Pre-formulated	Roche VENTANA BenchMark
PR	SP2	Pre-formulated	Roche VENTANA BenchMark
P16	MX007	Concentrated	Roche VENTANA BenchMark
CK5/6	MX040	Concentrated	Roche VENTANA BenchMark

Interpretation criteria:

• ER and PR immunoreactivity was evaluated in tumor cell nuclei using the Allred scoring system. The proportion score (PS) was assigned according to the percentage of immunoreactive tumor nuclei as follows: 0, no staining; 1, <1% positive cells; 2, 1–10% positive cells; 3, 11–33% positive cells; 4, 34–66% positive cells; and 5, 67–100% positive cells. The staining intensity score (IS) was graded as 0 (negative), 1 (weak), 2 (moderate), or 3 (strong). The final Allred score was calculated as the sum of PS and IS (range, 0–8). In this study, Allred scores were used as a semi-quantitative measure of ER and PR expression for exploratory comparison between ss-SCC and cervix-confined HSIL, rather than as a basis for therapeutic decision-making.
• Block-type p16 positivity was defined as strong and continuous nuclear and cytoplasmic staining involving the full thickness of the squamous epithelium affected by the lesion or a confluent area of invasive tumor. Patchy, focal, or discontinuous staining was interpreted as non-block-type expression. Block-type p16 staining was considered supportive of an HPV-associated squamous phenotype but was not regarded as evidence of tumor clonality or definitive proof of cervical origin.
• CK5/6 positivity was defined as cytoplasmic and/or membranous staining in lesional tumor cells and was interpreted only as an ancillary finding supporting squamous differentiation.

### 2.4 Literature search and study selection

A literature search was performed to identify previously reported cases of cervical ss-SCC and related superficial extension patterns. PubMed was searched from inception to December 2025 using combinations of terms related to cervical neoplasms, squamous differentiation, and superficial extension: (“Uterine Cervical Neoplasms”[MeSH] OR cervical neoplasm*[Title/Abstract] OR cervical cancer[Title/Abstract] OR cervical carcinoma[Title/Abstract]) AND (“squamous cell carcinoma”[Title/Abstract] OR HSIL[Title/Abstract] OR “high-grade squamous intraepithelial lesion”[Title/Abstract] OR “carcinoma in situ”[Title/Abstract]) AND (“superficial spread*”[Title/Abstract] OR “superficial extension”[Title/Abstract] OR “surface spread*”[Title/Abstract] OR “mucosal extension”[Title/Abstract] OR “contiguous spread”[Title/Abstract]). Eligible studies included original case reports and case series describing cervical squamous neoplasms with histologically documented superficial mucosal extension to contiguous genital-tract sites. Studies were excluded if they lacked original cases, described a non-cervical primary tumor, reported extra-cervical involvement attributable solely or predominantly to recurrent, metastatic, or conventional destructive invasive spread without histologically documented superficial mucosal continuity, lacked sufficient pathological information to establish cervical origin, or represented duplicate reports of previously published cases. When overlapping publications were identified, the most comprehensive report was selected. Reference lists of included studies were manually reviewed to identify additional eligible cases. The final literature cohort therefore comprised 25 publications describing 44 patients. Given the rarity of this pattern and the heterogeneity of published data, findings were summarized descriptively, and pooled quantitative analysis was not performed.

### 2.5 Statistical analysis

Statistical analyses were primarily descriptive because of the small cohort size (n=7). Categorical variables were summarized as numbers and percentages, and continuous variables were summarized as medians with ranges or means when appropriate. Clinical outcomes, including recurrence and survival status, were assessed at the last follow-up. Kaplan–Meier survival analysis and subgroup comparisons were not performed because the number of outcome events was insufficient for meaningful statistical modeling, with no deaths, only two recurrence events, and substantial heterogeneity in treatment approaches. The ER/PR comparison was considered exploratory and was summarized descriptively; inferential P values were not calculated. Statistical analyses were performed using SPSS version 20 (IBM Corp., Armonk, NY, USA).

## 3 Results

### 3.1 Clinical features of superficially spreading cervical squamous cell carcinoma

The cohort included seven patients aged 50–77 years (mean, 60 years). Five patients (71.42%) were aged 50–59 years. The age at first sexual intercourse ranged from 20 to 25 years, and the age at menopause ranged from 45 to 55 years. All patients had a history of childbirth; six (85.71%) had delivered one to three children, and one (14.29%) had delivered four or more children. The most common presenting symptom was postmenopausal bleeding (28.57%), followed by vaginal discharge (14.29%) and contact bleeding (14.29%). Three patients (3/7, 42.86%) were identified during routine Pap smear screening. Preoperative HPV testing detected HPV16 in six patients (85.71%) and HPV18 in one patient (14.29%). Preoperative SCC-Ag levels were normal in most patients (85.71%), with only one patient showing a mild elevation. Detailed demographics are summarized in **Table 1**. Radiologically, pelvic MRI generally showed only subtle abnormalities despite the histologically extensive superficial mucosal involvement. A fluid-distended endometrial cavity and a small cervical mass were occasionally observed. In some cases, a thin, uniform layer of early enhancement was observed on the endometrial surface, occasionally presenting with focal irregular thickening. Notably, deep invasion of the cervical stroma or myometrium by malignant cell nests was not identified on MRI (**Figure 1**).

**Table 1:** Demographic profile, clinical findings, and screening results.

Clinical elements	Number	Percentage
Age	n=7	
50-59	5	71.42
60-69	1	14.29
70-79	1	14.29
Clinical profile	n=7	
Postmenopausal bleeding	2	28.57
Vaginal discharge and pyometra	1	14.29
Contact bleeding	1	14.29
Routine Pap smear screening	3	42.86
Age at first sexual intercourse (years)	n=7	
≤20	0	0
21-25	7	100
Age at menopause (years)	n=7	
45-49	2	28.57
50-55	5	71.43
Childbearing history		
None	0	0
1-3	6	85.71
≥4	1	14.29
HPV type	n=7	
HPV 16	6	85.71
HPV 18	1	14.29
SCC-Ag	n=7	
Increased	1	14.29
Normal	6	85.71

**Figure 1:**
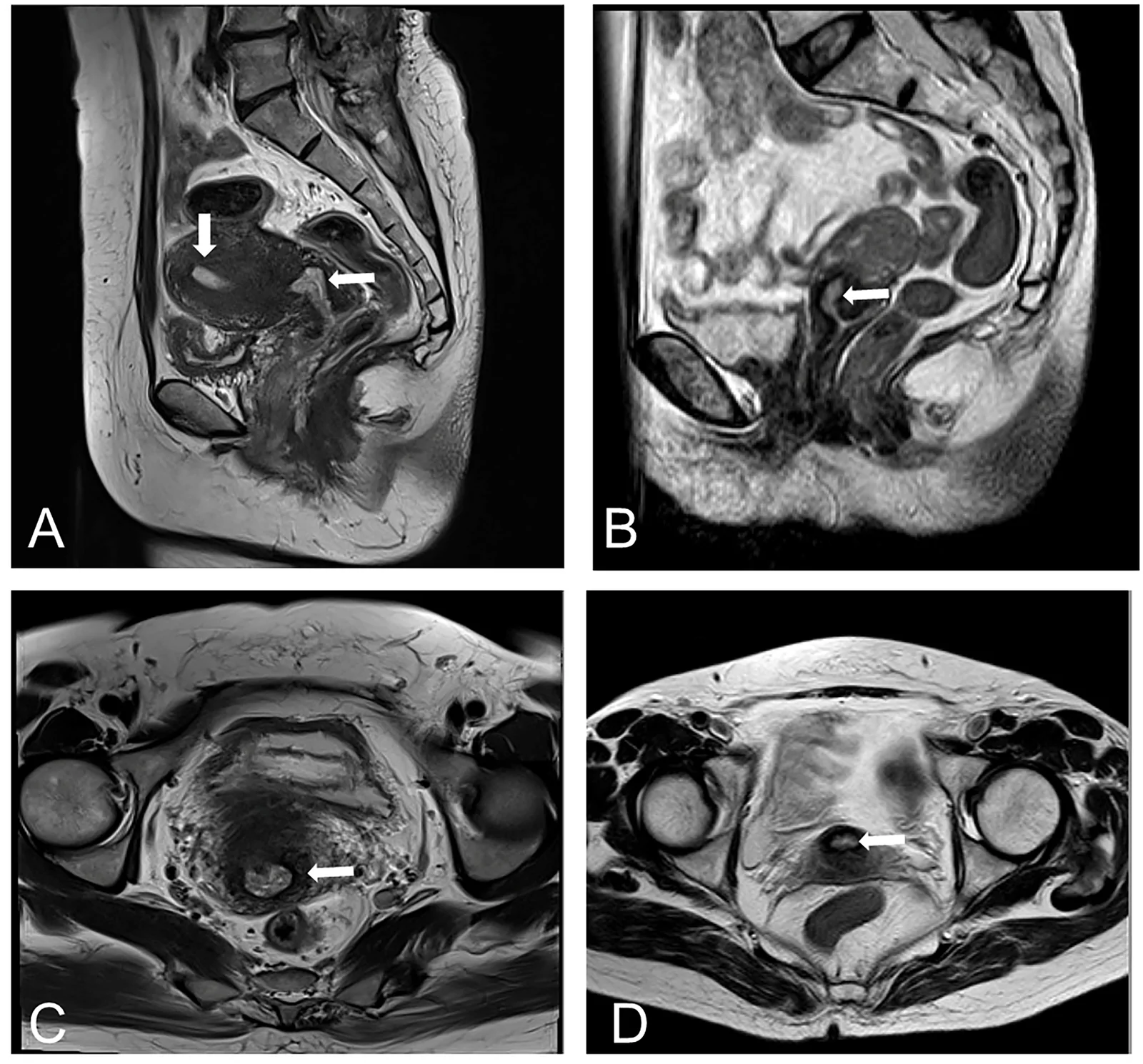
**(A)** Sagittal T2-weighted MRI section of patient No. 2, showing a fluid-distended endometrial cavity (vertical arrow) and a small mass (horizontal arrow) in the cervix. **(B)** Sagittal T2-weighted MRI section of patient No. 4, showing a small mass (horizontal arrow) in the cervix. **(C)** Axial T2-weighted MRI section of patient No. 2, showing a small lesion on the cervix (horizontal arrow); the cervical stromal ring appeared preserved, with no definite stromal invasion identified on MRI. **(D)** Axial T2-weighted MRI section of patient No. 4, showing a tiny lesion on the cervix (horizontal arrow); the cervical stroma ring was intact and the lesion did not invade the cervical stroma.

### 3.2 Histopathological characteristics

All seven cases fulfilled the predefined operational criteria for ss-SCC. All cases contained a limited invasive cervical SCC component, with a maximum stromal invasion depth of ≤5 mm; background HSIL was present in four cases. Despite limited stromal invasion, all cases demonstrated overt malignant squamous morphology with a superficial exophytic or papillary growth pattern. This architecture showed morphological overlap with the papillary SCC variant described in earlier classifications; however, papillary morphology alone was not considered diagnostic of ss-SCC. Instead, the defining feature was the presence of continuous, predominantly mucosal extension beyond the cervix. The combination of malignant cytological features and superficial spreading distribution supported classification within the cervical SCC spectrum despite limited stromal invasion.

In all cases, the lesion could be anatomically traced from the cervix along the mucosal epithelium into the endometrium, with additional involvement of the vagina or fallopian-tube mucosa in selected cases. Discrete invasive foci identified in the vagina, endometrium, fallopian tube, or ovary were documented separately because these represented additional invasive components rather than the defining pattern of superficial mucosal spread (**Figure 2**). All seven cases showed block-type p16 staining in both cervical and extra-cervical components, supporting an HPV-associated squamous phenotype; preoperative HPV testing identified HPV16 in six patients and HPV18 in one patient. Four patients had tumor involvement at the vaginal cuff margin.

**Figure 2:**
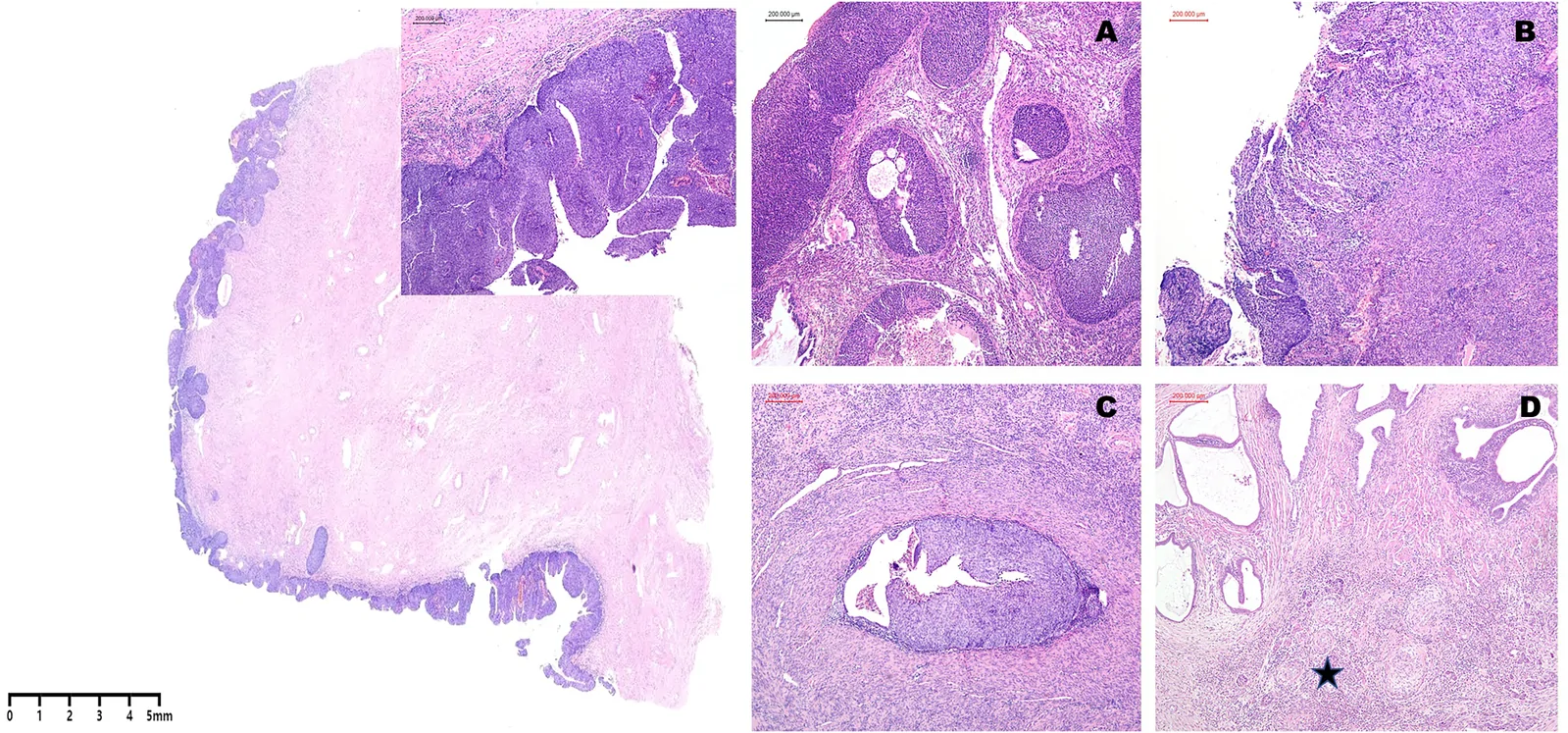
Anatomical distribution and invasive components of ss-SCC. The overview image shows a cervical-centered squamous lesion with continuous superficial extension along the genital tract mucosa to the lower uterine corpus and vaginal margin. The magnified view demonstrates papillary/exophytic architecture despite limited stromal invasion. **(A)** Continuous superficial involvement of the endometrial surface by the cervical squamous lesion. **(B)** Superficial endometrial extension accompanied by a discrete invasive focus within the uterine corpus. **(C)** Superficial mucosal extension involving the interstitial fallopian tube. **(D)** Additional tubo-ovarian involvement (asterisk) with tubal dilatation, architectural distortion, and adhesion to the ovary.

In Case 7, continuous extension along the tubal mucosa, concordant squamous morphology, block-type p16 expression, and CK5/6 positivity supported HPV-associated squamous differentiation and favored a cervical origin for the tubo-ovarian lesion (**Figure 3**). However, the anatomical interpretation of the involved sites requires careful distinction. The endometrial and tubal mucosal components could be explained by direct superficial extension along contiguous genital tract mucosa, whereas the ovarian involvement could not be attributed to this mechanism because the ovary lacks direct mucosal continuity with the cervical lesion. Instead, the ovarian invasive component, together with the presence of focal LVSI, suggested a potential conventional route of dissemination associated with more aggressive invasive behavior. Although LVSI does not establish the exact mechanism of ovarian involvement, this finding highlights that superficial mucosal extension and conventional invasive dissemination may coexist within the same case and should be evaluated separately (6).

**Figure 3:**
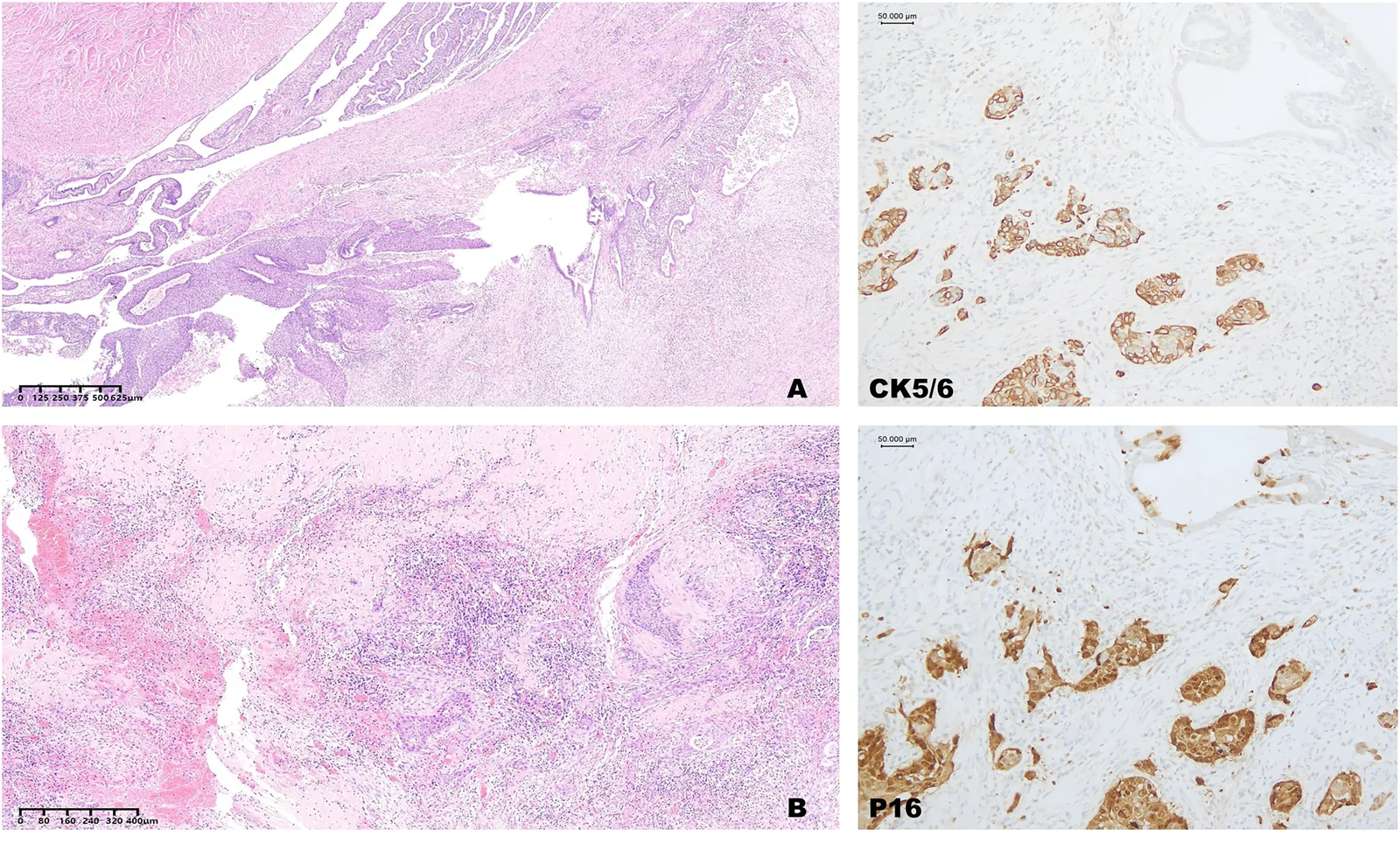
Histological and immunohistochemical evaluation of extra-cervical involvement in Case 7. Representative images show **(A)** continuous histological extension of the cervical squamous lesion along the fallopian-tube mucosa in a dilated and adherent tube, and **(B)** an invasive ovarian focus showing block-type p16 and CK5/6 expression, supporting an HPV-associated squamous phenotype. Cervical origin of the extra-cervical lesions was favored based on anatomical continuity and morphological concordance; however, p16 and CK5/6 expression alone do not establish site of origin or clonality.

### 3.3 Clinical management and follow-up

Treatment and follow-up data are listed in **Table 2**. Surgery was the primary treatment for all patients. Radical hysterectomy (Wertheim’s procedure) was performed in three patients (42.86%; stage IB2 or IIA1), while the remaining four patients (57.14%; stage IA1 or IA2) underwent total abdominal hysterectomy with bilateral salpingo-oophorectomy (TAH+BSO).

**Table 2:** Clinicopathological features, treatment, recurrence, and follow-up in the institutional cohort.

Patient no.	Surgery	FIGO stage	Cervical lesion	LVSI	Stromal invasion	Endometrium	Tubes and ovaries	Vaginal wall	Vaginal cuff	Adjuvant chemoradiotherapy	Recurrence	Follow-up (months)
F1	TAH+BSO	IA2	HSIL + SCC	Negative	Yes (≤5 mm)	SCC(<3mm)	Negative	HSIL	HSIL	YES	NO	17
F2	Wertheim’s	IIA1	SCC	Negative	Yes (≤5 mm)	HSIL	Negative	HSIL	HSIL	YES	NO	34
F3	Wertheim’s	IB2	SCC	Negative	Yes (≤5 mm)	HSIL	Negative	LSIL	Negative	NO	YES	48
F4	Conization followed by Wertheim’s	IIA1	SCC	Negative	Yes (≤5 mm)	SCC(<3mm)	Negative	SCC(<1mm)	HISL	YES	NO	11
F5	TAH+BSO	IA1	HSIL + SCC	Negative	Yes (≤5 mm)	HSIL	HSIL	HSIL	Negative	YES	NO	17
F6	TAH+BSO	IA1	HSIL + SCC	Negative	Yes (≤5 mm)	HSIL	Negative	HSIL	HSIL	NO	YES	30
F7	TAH+BSO	IA1	HSIL + SCC	Focal	Yes (≤5 mm)	HSIL	SCC; focal LVSI	Negative	Negative	YES	NO	54

TAH+BSO, total abdominal hysterectomy with bilateral salpingo-oophorectomy; HSIL, high-grade squamous intraepithelial lesion; LSIL, low-grade squamous intraepithelial lesion; SCC, squamous cell carcinoma; LVSI, lymphovascular space invasion. FIGO stage was assigned according to the 2018 FIGO staging system. In F3, staging was based on preoperative CT, which demonstrated a cervical mass measuring >2 cm.

Clinical follow-up was available for all seven patients, with a median follow-up duration of 30 months (range, 11–54 months). Five patients received postoperative adjuvant chemoradiotherapy. The two patients who did not receive initial adjuvant therapy developed local recurrence (2/7, 28.6%); both subsequently received salvage chemoradiotherapy and were alive without evidence of disease at the last follow-up. All seven patients were alive at last contact, and no deaths occurred during the follow-up period. The absence of death events and the limited number of recurrences precluded meaningful survival analysis or assessment of treatment-associated outcomes.

### 3.4 Literature review

The literature review identified 25 publications describing 44 patients who met the revised eligibility criteria (7–31). Reported patients were predominantly peri- or postmenopausal, similar to our cohort. Vaginal bleeding and abnormal discharge were the most frequent symptoms, while lower abdominal pain and pyometra were also reported. The endometrium was the most frequently involved extra-cervical site, followed by the fallopian tubes. Source reports commonly described cervical HSIL, often using historical terminology such as CIN or CIS, with or without limited microscopic stromal invasion. Superficial extension along the genital-tract mucosa was the predominant pattern, although limited invasive foci in the endometrium or fallopian tube were occasionally documented. Among cases with available follow-up, most patients were alive without evidence of disease, whereas one patient developed pulmonary metastasis (detailed in **Table 3**), indicating that the biological behavior of this growth pattern requires further clarification.

**Table 3:** Published cases meeting the revised literature-review eligibility criteria.

No.	Type (number if reported in series)	Age	Symptom	Cervical lesion	Extra-cervical site (i = invasion)	Surgery	Adjuvant therapy	Outcome
1 (13)	case	76	abdominal pain and pyometra	HSIL	Endometrium, fallopian tubes	TAH+BSO	Radiotherapy	NED
2 (14)	case	61	Incidental finding during screening	mi-SCC	Endometrium, fallopian tube, ovary	TLH+BSO	No	NED
3 (10)	case	45	postcoital bleeding with pelvic pain	HSIL + SCC	Endometrium, fallopian tubes	RAH+BS+L	Radiotherapy	NED
4 (9)	case series (15)	range 50–81	vaginal bleeding (most common,66.67%)	HSIL + SCC (12/15)	Endometrium (i), fallopian tube, ovary	NA	most were given chemoradiotherapy	NED
5 (15)	case	55	vaginal discharge	SCC	Endometrium	Wertheim’s hysterectomy	NA	NA
6 (16)	case	76	lower abdominal mass	SCC	Endometrium	TLH+BSO	Chemotherapy	Lung metastasis
7 (17)	case	46	vaginal bleeding and discharge	HSIL + SCC	Endometrium (i)	TAH+BSO+L	No	NED
8 (18)	case	60	persistent vaginal bleeding	mi-SCC	Endometrium (i)	Wertheim’s hysterectomy	NA	NA
9 (19)	case	62	vaginal bleeding	mi-SCC	Endometrium (i), fallopian tube (i)	Wertheim’s hysterectomy+BSO	NA	NA
10 (11)	case	57	Incidental finding during screening	HSIL	Endometrium (i)	TLH+BSO	Radiotherapy	NED
11 (8)	case	66	vaginal discharge	mi-SCC	Endometrium	TLH+BSO	No	NED
12 (20)	case	63	postmenopausal bleeding	SCC	Endometrium	Wertheim’s hysterectomy+BSO	NA	NED
13 (21)	case	53	Incidental finding during screening	HSIL	Endometrium	TAH+BSO	NA	NA
14 (22)	case	66	Incidental finding during screening	HSIL	Endometrium (i)	TAH	NA	NA
15 (23)	case	67	lower abdominal pain	HSIL + mi-SCC	Endometrium, fallopian tubes, Additional involvement:ovaries; omentum (i)	Wertheim’s hysterectomy +BSO+omentectomy+ transverse colon resection	Chemotherapy	NED
16 (24)	case	45	vaginal bleeding, lower abdominal pain	mi-SCC	Endometrium	TAH+BSO	NA	NA
17 (25)	case	72	abdominal mass	mi-SCC	Endometrium	TAH+BSO	No	NED
18 (12)	case series (2)	64	vaginal bleeding	mi-SCC SCC	Endometrium, vagina (i) Endometrium	TH+BSO	NA	NED
		59						
19 (26)	case series (3)	65	abnormal screening	SCC	Endometrium	Wertheim’s hysterectomy	NA	NA
		60	vaginal bleeding and discharge	SCC	Endometrium (i)	Wertheim’s hysterectomy		
		49	vaginal bleeding	HSIL	Endometrium, vaginal surface	TH+BSO		
20 (27)	case	53	vaginal bleeding	mi-SCC	Endometrium (i), fallopian tubes, Additional involvement: ovaries	Wertheim’s hysterectomy+BSO	NA	NED
21 (28)	case	76	NA	HSIL	Endometrium, fallopian tubes, Additional involvement: ovaries	NA	NA	NA
22 (29)	case	70	vaginal discharge	mi-SCC	Endometrium	TAH+BSO	No	NED
23 (30)	case	52	vaginal stenosis and pyometra	HSIL	Endometrium	Wertheim’s hysterectomy+BSO	NA	NED
24 (31)	case	55	vaginal bleeding and discharge	SCC	Endometrium (i)	Wertheim’s hysterectomy	NA	NA
25 (7)	case series (3)	70	NA	SCC	Endometrium, vaginal surface	Wertheim’s hysterectomy	No	NED
		44	abnormal screening	HSIL	Endometrium	Extrafascial hysterectomy	No	NED
		67	vaginal bleeding	HSIL	Endometrium, fornix	TAH+BSO	No	NED

TH, total hysterectomy; TLH, total laparoscopic hysterectomy; RAH, radical abdominal hysterectomy; TAH+BSO, total abdominal hysterectomy with bilateral salpingo-oophorectomy; L, lymphadenectomy; BS, bilateral salpingectomy; HSIL, high-grade squamous intraepithelial lesion; SCC, squamous cell carcinoma; mi-SCC, microinvasive squamous cell carcinoma; NED, no evidence of disease; NA, not available; NR, not reported. Historical CIS/CIN3 terminology was harmonized to HSIL.

### 3.5 Comparison of ER and PR expression

Given the predominance of peri- and postmenopausal patients in both our cohort and previously reported cases, ER and PR immunohistochemistry was performed as an exploratory assessment of whether tumor-cell hormone receptor expression might represent a biological correlate of this distinctive superficial spreading pattern. Using the prespecified Allred scoring system, both the ss-SCC group (n=7) and the cervix-confined HSIL comparison group (n=5) showed reduced or absent tumor-cell nuclear ER/PR expression, without an apparent qualitative difference between the groups. No formal statistical comparison was performed because of the small, unmatched groups. Representative ER and PR staining patterns are shown in **Figure 4**.

**Figure 4:**
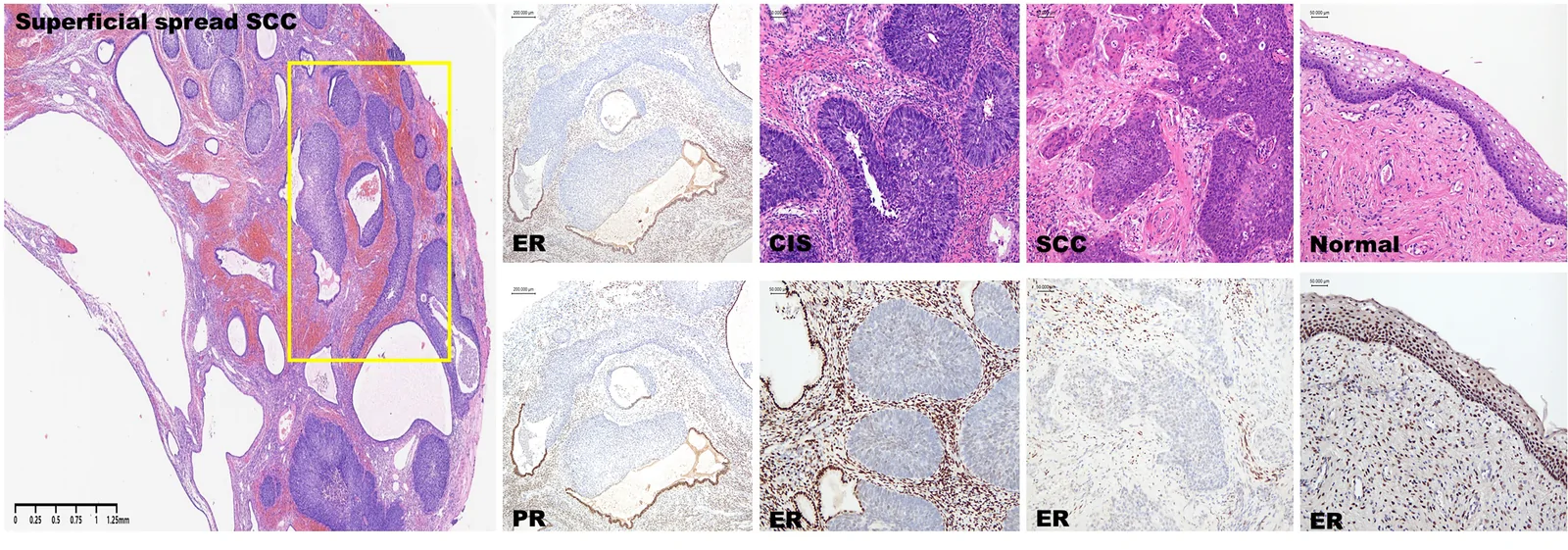
Exploratory ER/PR immunohistochemical assessment. Representative images show ss-SCC involving the epithelial surface and glands within an endometrial polyp, together with cervix-confined HSIL, invasive SCC, and non-neoplastic cervical squamous epithelium used as comparison tissues. Tumor-cell nuclear ER and PR expression was absent or markedly reduced in ss-SCC and cervix-confined HSIL or SCC cases.

## 4 Discussion

### 4.1 Clinical context

The clinical profile of our cohort was similar to that described in the 44 eligible published cases: patients were predominantly peri- or postmenopausal, and postmenopausal bleeding, abnormal discharge, and pyometra were recurring presentations (8–11). Cervical atrophy or stenosis may contribute to pyometra and delayed recognition, but the available data do not establish a causal age- or hormone-dependent mechanism. Biopsy and conization may also underestimate disease extent because they do not sample the upper genital tract. Thus, in an older patient with cervical HSIL or a microscopically detected invasive SCC and unexplained uterine-cavity findings, additional endometrial evaluation and pelvic imaging may be considered.

### 4.2 Radiological findings

MRI in our cohort showed small cervical lesions, occasional distension of the endometrial cavity, and a thin or focally thickened enhancing endometrial surface, while a deeply destructive cervical or myometrial mass was not identified. Published reports likewise describe subtle endometrial thickening, continuous enhancement, or intrauterine mural nodules (9, 16). These findings may alert the multidisciplinary team to upper-tract involvement, but a thin mucosal lesion can remain radiologically occult; histological sampling is therefore required to determine continuity and invasion.

### 4.3 Histopathological diagnosis and ancillary studies

The defining feature of ss-SCC is a cervical-centered squamous lesion that can be anatomically traced continuously along the genital tract mucosa, with extra-cervical involvement of anatomically contiguous mucosal surfaces showing a predominantly superficial growth pattern. Although all seven cases demonstrated papillary and/or exophytic architecture and therefore showed morphological overlap with the papillary SCC variant recognized in the 2014 WHO classification, papillary architecture alone does not adequately capture the extensive mucosal distribution characteristic of ss-SCC. As ss-SCC is not currently recognized as a distinct diagnostic category in the WHO Classification of Female Genital Tumors, we propose that the term ss-SCC should be used as a descriptive growth pattern rather than a separate tumor entity within the HPV-associated cervical SCC spectrum. Histological diagnosis should continue to follow current WHO terminology, with HSIL used for non-invasive squamous lesions and invasive SCC classified according to the presence and measured depth of stromal invasion. All patients had preoperative cervical HPV testing demonstrating HPV16 or HPV18 infection, and all seven cases showed block-type p16 staining in both cervical and extra-cervical components, supporting an HPV-associated squamous phenotype. CK5/6 immunoreactivity supported squamous differentiation in Case 7. However, neither p16 nor CK5/6 is site-specific, and p16/HPV concordance alone cannot establish clonality or prove that anatomically separated lesions share a common cervical origin. In this cohort, cervical origin was primarily assigned based on demonstrable anatomical continuity and morphological concordance between cervical and extra-cervical components. HPV *in situ* hybridization across involved sites or molecular clonality analysis would provide stronger support when continuity is uncertain.

### 4.4 Comparison with previous reports and diagnostic implications

Compared with previous reports, our study provides several additional contributions. First, prior descriptions have used heterogeneous terminology, including superficial extension of CIN/CIS and superficial spreading SCC, reflecting the absence of standardized diagnostic criteria. In contrast, we propose an operational framework based on cervical-centered origin, demonstrable anatomical continuity, predominant superficial mucosal extension, morphological concordance, and exclusion of alternative primary or metastatic processes. Second, while previous reports often emphasized the extent of anatomical involvement, our study further distinguishes the defining superficial mucosal spreading component from separate invasive or disseminated components, particularly in non-contiguous sites such as the adnexa or ovary. Third, our study integrates pathological classification with current WHO terminology and FIGO/AJCC staging principles, emphasizing that ss-SCC should be interpreted as a descriptive growth pattern rather than a separate diagnostic entity. These findings extend previous observations by providing a more reproducible framework for diagnosis, reporting, and clinical interpretation.

### 4.5 Staging, treatment, and follow-up

Superficial extension beyond the cervix is not recognized as a separate category in the 2018 FIGO staging system or the eighth edition of the AJCC staging manual. Therefore, cases with ss-SCC should be staged according to established anatomical criteria for cervical SCC, while the pattern, route, and extent of superficial extension should be explicitly documented. Although the present series cannot determine whether limited mucosal involvement should independently influence staging, the observed clinical outcomes, together with previously reported cases, raise the possibility that superficial mucosal dissemination along anatomically contiguous genital tract surfaces may represent a clinically distinct pattern from conventional destructive stromal invasion or hematogenous dissemination. The combined observations from our cohort and published literature indicate that adverse clinical outcomes have not been consistently observed in cases characterized by predominant superficial extension along anatomically contiguous mucosal surfaces or only minimal stromal invasion. However, these observations should be interpreted cautiously, given the small sample sizes, heterogeneous treatment approaches, variable follow-up durations, and limited number of outcome events, which preclude robust conclusions regarding prognosis.

### 4.6 Potential biological mechanisms underlying ss-SCC

The biological mechanisms underlying ss-SCC remain incompletely understood. Although direct evidence is limited, selected studies have demonstrated clonal relationships between cervical and extra-cervical genital tract lesions using loss-of-heterozygosity analysis or HPV-based approaches, supporting the concept that at least a subset of cases represents genuine contiguous tumor spread rather than independent multifocal disease (32). The frequent occurrence of ss-SCC among postmenopausal women prompted an exploratory assessment of whether altered hormone receptor expression might represent a potential biological correlate of this growth pattern. ER and PR immunohistochemistry was therefore performed to compare hormone receptor expression between ss-SCC and cervix-confined HSIL. The absence of a consistent difference between groups does not support ER/PR loss as a specific biological driver, diagnostic feature, or defining mechanism of ss-SCC.

### 4.7 Study limitations

This study has several limitations. First, the retrospective single-center design and small cohort size reflect the rarity of this growth pattern but also limit the ability to fully evaluate prognostic factors and may introduce potential referral, selection, and sampling biases. Second, treatment approaches were heterogeneous and follow-up duration was relatively limited; therefore, the study was not powered to perform robust survival analyses or determine the independent impact of specific therapeutic strategies. Molecular clonality analysis was not performed across all involved anatomical sites, and evidence supporting a common origin was therefore primarily based on anatomical continuity, morphological concordance, and HPV-associated features. ER/PR assessment was exploratory and performed in a limited number of cases; these findings should be interpreted as hypothesis-generating rather than as evidence for a diagnostic or prognostic biomarker. Finally, published literature consists largely of case reports and small series with variable terminology, incomplete follow-up, and potential publication bias. Future multicenter studies with standardized diagnostic criteria, longer follow-up, and integrated molecular analyses are needed.

## 5 Conclusions and take-home messages

- ss-SCC should be recognized as a rare histological growth pattern within the HPV-associated cervical SCC spectrum, characterized by a cervical-centered malignant squamous lesion with predominantly continuous superficial extension along the mucosal surfaces of the genital tract. Although not currently recognized as a separate WHO diagnostic category, ss-SCC represents a reproducible pattern that warrants specific pathological recognition.
- A reproducible diagnosis requires confirmation of cervical origin, demonstration of anatomical and histological continuity, and exclusion of an independent extra-cervical primary tumor. HPV genotyping or molecular clonality analysis may provide additional supportive evidence when anatomical continuity is incomplete or uncertain.
- Extra-cervical involvement in ss-SCC, particularly when confined to continuous superficial mucosal extension or associated with minimal stromal invasion, does not necessarily indicate conventional metastatic dissemination or uniformly adverse outcome. However, due to limited available evidence, the prognostic significance of ss-SCC and the need for adjuvant chemoradiotherapy cannot yet be definitively established.
- Because ss-SCC is not currently defined as a distinct category in existing diagnostic or staging systems, pathology reports should explicitly document the extent and distribution of superficial extension, the relationship between involved anatomical sites, cervical and extra-cervical invasion depth, LVSI, margin status, and any non-contiguous invasive components to facilitate multidisciplinary clinical decision-making.

## Data Availability

The original contributions presented in the study are included in the article/supplementary material. Further inquiries can be directed to the corresponding author.
